# Medical Statistics of the U. S. Army, for 21 Years

**Published:** 1860-11

**Authors:** 


### Medical Statistics of U. S. Army for 21 Years.

We are in receipt, through our Representatives in Congress, of the report of Secretary Jefferson Davis, of the War Department, to the Senate of the United States, "On the Sickness and Mortality in the Army of the United States," from the first of January 1839 to the first of January 1855; and also a continuation of the same from the last named period to January first 1860. By Hon. John B. Floyd, Secretary of War. These reports were compiled from the records of the Surgeon General's office, under the direction of Brevet Brigadier, General Thomas Lawson, *Surgeon General of the United States Army*, by Richard H. Coolidge, M. D., *Assistant Surgeon of the United States Army.*

The documents before us, giving a statistical account of the "Sickness and Mortality" of the Army stationed at the various points on the sea-coast and the interior—in Northern and Southern latitudes—in sterile regions and rich vallies, are exceedingly interesting to the medical man.

The stations from which the reports are obtained, are arranged into the Northern, Middle and Southern divisions of the Army; and statistical tables of Sickness and Mortality exhibit separately, fevers, diseases of the digestive system, and diseases of the respiratory system, as they have been found to accur at the various posts in the respective divisions.

From these tables something reliable may be obtained in regard to the difference in the nature and prevalence of disease in localities differing from each other in point of climate and other respects. The Surgeons from whom the reports are originally derived, stationed at the several points, being educated, intelligent and reliable men, there is certainty in the correctness of the statements; and the uniformity in the the habit, occupation and manner of living of the subjects of disease, prevents the erroneous conclusions, as respects the true causes of difference to which we are liable to arrive, when such uniformity does not obtain. With these advantages, we think no mode of study is more satisfactory and profitable, than statistical investigation.

In addition to the proportional number diseased, the character of disease and the mortality, accurate discription of the treatment instituted, by each Surgeon in charge, is given in these reports; so that the investigator may determine with some certainty, by comparison of the tables, what plan of treatment has proved most successful.

It is interesting and profitable to ascertain how far the generally received opions of the prevalence and mortality of certain diseases in particular latitudes &c. are sustained by these records. The following consolidated tables are copied for information on this subject:

*Consolidated Table exhibiting the amount and annual ratio of Sickness and Mortality for each region from Fevers.*

| No. | Regions. | Mean Strength. | No. Treated. | D'ths | Prop'n of de'ths to cases. | Ratio ca'es pr 1,000 of m'n st'gth. |
|---|---|---|---|---|---|---|
| 1 | Coast of New England,........ .. .......... | 4,529 | 566 | 2 | 1 in 283 | 125 |
| 2 | Harbor of New York,.................... | 12,856 | 3,307 | 50 | 1 in 66 | 256 |
| 3 | West Point,.................. ........... | 9,400 | 1,221 | 3 | 1 in 407 | 130 |
| 4 | North Interior, East,.. .................. | 3,553 | 384 | 3 | 1 in 128 | 108 |
| 5 | The Great Lakes, ...................... | 10,770 | 3,503 | 22 | 1 in 159 | 325 |
| 6 | North Interior, West,......... ........... | 13,892 | 4,106 | 17 | 1 in 241 | 295 |
| 7 | Middle Atlantic,*.. ..................... | 8,407 | 2,513 | 45 | 1 in 56 | 299 |
| 8 | Middle Interior, East,.................... | 3,804 | 1,103 | 8 | 1 in 138 | 289 |
| 9 | Newport Barracks, Kentucky,. .......... | 2,475 | 1,593 | 9 | 1 in 177 | 643 |
| 10 | Jefferson Barracks and St. Louis Arsenal,.. | 6,956 | 6,114 | 54 | 1 in 113 | 879 |
| 11 | Middle Interior, West,........... .... | 9,681 | 8,629 | 27 | 1 in 319 | 890 |
| 12 | South Atlantic,.......................... | 3,401 | 1,080 | 44 | 1 in 25 | 317 |
| 13 | South Interior, East,.................... | 6,419 | 4,460 | 90 | 1 in 50 | 694 |
| 14 | South Interior, West,.................... | 12,312 | 15,910 | 44 | 1 in 362 | 1,292 |
| 15 | Atlantic Coast of Florida,... ............ | 1,835 | 1,625 | 21 | 1 in 77 | 885 |
| 16 | Interior, and Gulf Coast of Florida,....... | 3,939 | 8,073 | 39 | 1 in 207 | 2,049 |
| 17 | Texas, Southern Frontier, ............... | 7,310 | 8,911 | 133 | 1 in 67 | 1,219 |
| 18 | Texas, Western Frontier,................. | 13,183 | 11,387 | 20 | 1 in 569 | 863 |
| 19 | New Mexico,.... .. .................... | 13,445 | 5,725 | 30 | 1 in 191 | 425 |
| 20 | California, Southern,..................... | 3,865 | 699 | 13 | 1 in 54 | 181 |
| 21 | California, Northern,..................... | 3,853 | 3,433 | 22 | 1 in 156 | 890 |
| 22 | Oregon and Washington,.................. | 8,974 | 2,117 | 4 | 1 in 529 | 236 |
| 23 | Utah, .................................. | 5,842 | 1,206 | 14 | 1 in 86 | 206 |

*This includes cases of yellow fever brought to Fort Monroe from Vera Cruz, in 1848.

---

*Consolidated Table exhibiting the amount and annual ratio of sickness and mortality in each region from Phthisis Pulmonalis.*

| No. | Regions. | Mean str'gth | No. t'ed. | D'ths | Prop'n of Deaths to Cases. | Ratio cas's pr 1,000 of m'n st'gth. |
|---|---|---|---|---|---|---|
| 1 | Coast of New England,...... | 4,529 | 21 | 6 | 1 in 3.5 | 4.6 |
| 2 | Harbor of New York,......... | 12,856 | 73 | 40 | 1 in 1.8 | 5.6 |
| 3 | West Point,................. | 9,400 | 14 | 14 | 1 in 1 | 1.5 |
| 4 | North Interior, East,.......... | 3,533 | 17 | 10 | 1 in 1.7 | 4.7 |
| 5 | The Great Lakes,.............. | 10,770 | 49 | 34 | 1 in 1.4 | 4.5 |
| 6 | North Interior, West,.... ... | 13,892 | 44 | 27 | 1 in 1.6 | 3.1 |
| 7 | Middle Atlantic,.............. | 8,407 | 28 | 19 | 2 in 3 | 3.3 |
| 8 | Middle Interior, East,......... | 3,804 | 11 | 6 | 1 in 1.8 | 2.8 |
| 9 | Newport Barracks, Kentucky, | 2,475 | 9 | 6 | 1 in 1.5 | 3.6 |
| 10 | Jefferson Barracks & St. Louis Arsenal,............... | 6,956 | 26 | 24 | 1 in 1.1 | 3.7 |
| 11 | Middle Interior, West,........ | 9,681 | 36 | 21 | 1 in 1.7 | 3.7 |
| 12 | South Atlantic,............... | 3,401 | 27 | 6 | 1 in 4.5 | 7.9 |
| 13 | South Interior, East,...... ... | 6,419 | 44 | 29 | 1 in 1.5 | 6.9 |
| 14 | South Interior, West,......... | 12,312 | 41 | 28 | 1 in 1.5 | 3.3 |
| 15 | Atlantic Coast of Florida,.... | 1,835 | 5 | 2 | 1 in 2.5 | 2.7 |
| 16 | Interior & Gulf Coast of Fla.,. | 3,939 | 31 | 6 | 1 in 5 | 7.8 |
| 17 | Texas, Southern Frontier,.... | 7,310 | 24 | 16 | 1 in 1.5 | 3.3 |
| 18 | Texas, Western Frontier,..... | 13,183 | 48 | 22 | 1 in 2.2 | 3.6 |
| 19 | New Mexico,................ | 13,445 | 25 | 4 | 1 in 6.2 | 1.8 |
| 20 | California, Southern,.......... | 3,865 | 19 | 9 | 1 in 2.1 | 4.9 |
| 21 | California, Northern,.......... | 3,853 | 21 | 9 | 1 in 2.3 | 5.4 |
| 22 | Oregon and Washington...... | 8,974 | 45 | 10 | 1 in 4.5 | 5 |
| 23 | Utah,...................... | 5,842 | 8 | 1 | 1 in 8 | 1.3 |

